# 3D human epithelial models: a promising platform for studying *Candida* infections and novel antifungal therapeutic options

**DOI:** 10.3389/fcimb.2025.1680261

**Published:** 2025-09-03

**Authors:** Cristina Schöpf, Florentine Marx

**Affiliations:** Institute of Molecular Biology, Biocenter, Medical University of Innsbruck, Innsbruck, Austria

**Keywords:** 3D human epithelial models, reconstructed human epidermis, full-thickness skin model, *Candida*, infection, antifungal therapy

## 1 Introduction

*Candida albicans* is a common commensal organism found in approximately 50% of the population, colonizing various body sites including the gastrointestinal and reproductive tracts, and oral cavity. While typically harmless, the tightly regulated coexistence can be disturbed by various factors like antibiotics, stress, and underlying health conditions, and can result in massive overgrowth that potentially evolves into symptomatic epithelial candidiasis.

*C. albicans* is responsible for a wide range of infections, including vulvovaginal candidiasis (affecting 75% of women), diaper rash (in 50% of children under 12 months), oral thrush (in 30-80% of patients with underlying diseases), and infections (second most common fungal infection in dermatology). This microorganism is particularly significant in medical settings, causing 50% of nosocomial infections through medical device transmission. It can develop into invasive or disseminated forms with high morbidity and mortality, making it one of the most lethal opportunistic fungal pathogens in humans. Additionally, there’s a growing trend of non-albicans *Candida* species (NACs) like *Candida parapsilosis*, *Candida tropicalis*, and others, which are increasingly observed worldwide. The emergence of these multi-resistant fungal pathogens, like *Candida auris*, presents significant medical challenges (Brown et al., 2012; Denning et al., 2018; Nobile and Johnson, 2015; Taudorf et al., 2019).

The study of *Candida* infections in humans, particularly those affecting epithelial surfaces, has long been constrained by the limitations of traditional *in vitro* and animal models. Since 1959, when Russel and Burch first defined the 3Rs (reduction, refinement and replacement) of animal models (Russell and Burch, 1959), major efforts have been put into the advancement of biomimetically and physico-chemically accurate tissue equivalents. An important step forward in this regard has been the introduction of three-dimensional (3D) human epithelial models, which in the last decades have emerged as sophisticated alternatives that bridge the gap between simplified cell culture systems and complex *in vivo* conditions.

Some researchers still express skepticism regarding the complexity and costs associated with these *in vitro* models. However, we assert that 3D human epithelial models, including those representing the human, are highly effective and straightforward ethical alternatives to animal models, and are rapidly becoming indispensable for meaningful antifungal research.

This opinion paper examines the current state of 3D epithelial models, focusing specifically on models in *Candida* research and their potential impact on antifungal drug development.

## 2 Advantages of 3D reconstructed models for *Candida* research

### 2.1 Physiological relevance

**Figure 1:**
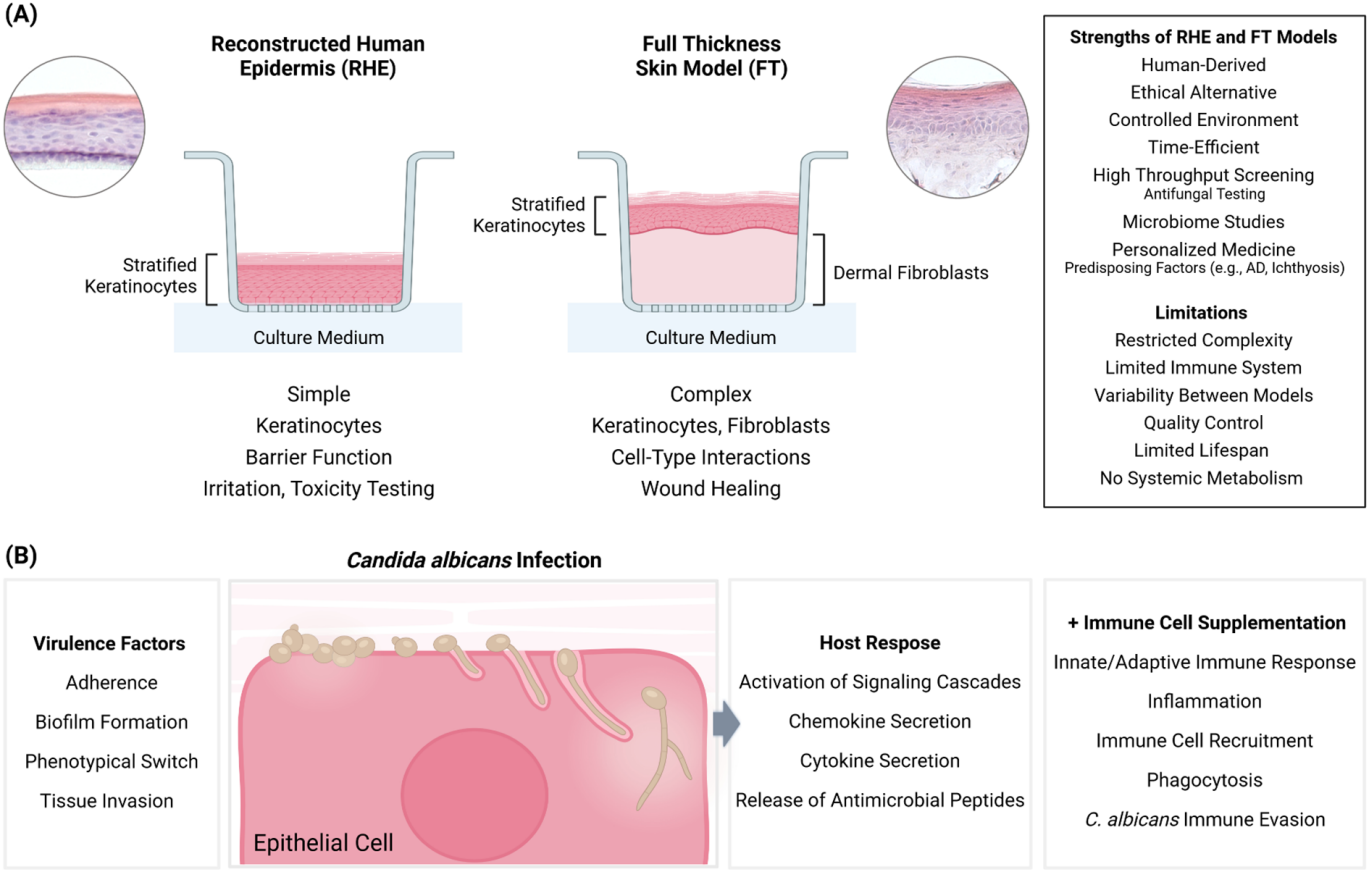
Advantages, limitations of RHE and FT skin models, and their versatility in studying *C. albicans* Infection. **(A)** Schematic representation of RHE and FT skin models cultured at ALI in transwells. Representative hematoxylin and eosin (H&E)-stained tissue sections are displayed for RHE (left circle) and the FT skin model (right circle). Key structural features and primary fields of application are illustrated below, while general strengths and limitations are summarized in the box on the right. AD, atopic dermatitis. **(B)** Key virulence factors of *C. albicans* during infection of 3D tissue models are summarized in the left box. A schematic representation of the infection process is depicted in the center. The box on the right outlines the corresponding and measurable host responses, while the far-right box lists additional analytical parameters when tissue models are supplemented with immune cells. Created with BioRender.com.

In our view, simple cell culture models have led us down numerous dead ends in understanding processes related to *Candida* infection and the development of antifungal treatment options. 3D reconstructed epithelial models have the potential to address this fundamental problem. For example, 3D models that reproduce the human *in vitro* include reconstituted human epidermis (RHE) or full thickness (FT) models, which exhibit distinct characteristics in their epidermal and full-thickness configurations (**Figure 1A**). RHE models are composed exclusively of human keratinocytes, which are seeded into transwells, and cultivated under submerged conditions prior to transitioning to the air-liquid interface (ALI). When cultivated under ALI conditions, the model acquires a stratified epithelial structure comprising distinct basal, spinous, granular, and cornified layers. These models develop a competent barrier function with an appropriate lipid composition and form functional tight junctions and desmosomes. They also express differentiation markers (e.g., involucrin, filaggrin, and keratins). The simplicity and limited physiological complexity of RHE models restrict their potential for investigating cell-type interactions. However, they provide a direct visualization of early *Candida* infection processes, an assessment of pathogen invasion mechanisms, and an evaluation of host cellular processes in response to both *Candida* colonization and antifungal drug application (Mewes et al., 2007; Suhail et al., 2019) (**Figure 1B**).

In contrast, FT models integrate both the epidermal and dermal components in order to provide a more comprehensive simulation of the native human skin architecture (**Figure 1A**). A fully differentiated epidermis is developed on dermal fibroblasts, which are typically seeded on natural or synthetic scaffolds, such as collagen or polymerized hydrogels, respectively. FT models facilitate more sophisticated research into cell-cell interactions, wound healing processes, and complex infection dynamics across the epidermal and dermal layers. The complexity of FT – and even RHE – models can be further enhanced by integrating immune cells, like Langerhans cells, macrophages or T cells, to investigate immune responses and inflammatory mechanisms (Jia and Atwood, 2024; Mewes et al., 2007; Rademacher et al., 2018; Suhail et al., 2019) (**Figure 1B**).

### 2.2 Experimental versatility

3D models can be used to examine host-pathogen interactions and determine virulence factors that underpin *Candida* infections. These include epithelial adhesion and invasion, biofilm formation, the mechanisms underlying morphological switching, the progression of host cell damage through the secretion of hydrolytic enzymes, and inflammatory and immunomodulatory response cascades that are induced upon contact between the yeast pathogen and the host (**Figure 1B**). The versatility of 3D epithelial models, including intestinal, oral, and vaginal epithelia, has been validated in several studies, demonstrating their utility in studying the infection mechanism caused by *Candida* spp (Boero et al., 2021; Mills and Estes, 2016; Naglik et al., 2017; Schaller et al., 2002). However, research on *Candida* infection in human skin models remains limited (Holzknecht et al., 2022). Addressing this gap is crucial to advancing our understanding of fungal infections in skin tissues. Significant insights into the mechanistic framework of fungal epithelial infections and host responses have primarily been derived from reconstructed two-dimensional (2D) and 3D mucosal systems, which predominantly mimic the oral epithelium. These models have been instrumental in elucidating the infection process, which involves epithelial adhesion, the morphological transition of *Candida* from spherical yeast cells to hyphal forms, and subsequent epithelial invasion and dissemination through active penetration and endocytosis. For instance, studies using these models have revealed the upregulation of key virulence factor-encoding genes in *C. albicans*. Examples include the *EED1* gene, which is essential for maintaining hyphal elongation and enabling deep epithelial penetration; the *ALS3* gene, which encodes the agglutinin-like sequence 3 protein that promotes endocytosis in stratified epithelia; and the *ECE1* gene, which encodes the precursor for candidalysin, a toxin that damages epithelial membranes and activates a pro-inflammatory host response (Zakikhany et al., 2007). From the host’s perspective, studies using 3D reconstructed oral epithelium have provided valuable insights into the activation of signaling cascades, such as the NF-κB, MAPK, and PI3K/Akt-mTOR pathways. These pathways play a critical role in triggering immune responses and protecting epithelial cells from *C. albicans* -induced damage (Moyes et al., 2014). This body of research underscores the importance of further developing and utilizing advanced epithelial models to deepen our understanding of fungal infections and host-pathogen interactions.

Although cell-based models, such as monolayers and 2D models, are relatively simple, they have failed to predict therapeutic success. In contrast, evidence suggests that animal models often produce misleading results, and clinical trials based on traditional animal models have high failure rates. Consequently, 3D models capable of replicating a *Candida* infection offer distinctive advantages in the development of innovative therapeutic agents for topical administration. Such models provide sophisticated platforms for high-throughput antifungal screening, assessment of novel delivery systems, evaluation of combination therapies and studies of resistance mechanisms (**Figure 1A**). The objective of these studies is to identify novel antifungal candidate molecules that demonstrate improved efficacy, superior tissue penetration, and minimal host toxicity. These tools are optimal for assessing drug penetration and permeation through discrete epithelial layers, and – last but not least – studying the interaction between drug, host and pathogen, which facilitates the drawing of conclusions regarding the safe applicability and curative efficacy of an antifungal drug. The value of 3D models in evaluating novel antifungal compounds under conditions that closely mimic human infection scenarios is particularly highlighted in studies such as those performed by (Fleischli et al., 2015; Holzknecht et al., 2022; Liang et al., 2023; Salminen et al., 2023; Schäfer-Korting et al., 2008; Suhail et al., 2019). These models also facilitate the optimization of dosing regimens for new antifungals, which in turn leads to modified clinical trial protocols and improved patient outcomes.

### 2.3 Reproducibility and standardization

3D models can either be constructed individually in a laboratory setting according to published protocols, or be purchased from a specialized company, which offers several advantages over traditional methods. The standardized production of commercial models ensures a batch-to-batch consistency and a continuous availability. The utilization of well-defined growth conditions and medium composition, in conjunction with the implementation of sophisticated infection protocols within research laboratories further guarantee the elaboration of highly reproducible data and quantifiable endpoints for analysis (Mewes et al., 2007; Poumay and Coquette, 2007).

### 2.4 Cost-effective alternative

The long duration and high costs associated with preclinical studies in animal models, coupled with the discrepancies observed in the therapeutic response between animal models and patients, serve to further complicate the search for new antimicrobial compounds. The use of 3D models has been demonstrated to result in a reduction in the variability of experimental outcomes when compared to those observed in animal studies. Furthermore, the use of a substantial number of animals for the high throughput screening of potential candidate molecules for their efficacy, delivery, and safe applicability is constrained by ethical, regulatory, practical or economic considerations during the initial stages of drug development. 3D models facilitate higher-throughput screening, thereby accelerating the search for effective antifungal drugs and reducing the number of animals sacrificed, thus lowering expenses, while increasing the accuracy of drug efficacy predictions.

## 3 Discussion

### 3.1 Limitations and challenges

Current challenges encountered in manufacturing 3D models are (i) relatively high costs that depend on the complexity of the models; (ii) high quality control requirements; (iii) limited shelf-life, and (iv) storage and transport constraints. Furthermore, the source of human cells (keratinocytes and fibroblasts) and variations in culture conditions may affect model reproducibility. It is noteworthy that some companies that provide ready-to-use models, including culture media for cultivation of these models, do not disclose the composition of the medium. This could hinder the interpretation of the data if certain compounds have the potential to influence the experimental outcome. Researchers encounter technical challenges when experimental approaches necessitate long-term maintenance, infection control procedures, and complex data analysis. The aforementioned obstacles have led to the identification of key variables that affect model performance. Consequently, new guidelines have been introduced for standardization of protocols employed in industry, and for quality control and experimental design in the research sector. One example for this is the implementation of guidelines for *in vitro* skin irritation testing of chemicals and compounds for topical application (Organization for Economic Cooperation and Development [OECD], 2020).

In addition to these technical considerations, researchers are confronted with a number of biological limitations. 3D skin models are unable to fully replicate the vascular networks, neural components, systemic responses or complex tissue interactions. The absence of resident and circulating immune cells precludes the study of adaptive or systemic immune responses to the colonization with *C. albicans*, the formation of biofilm and the hyphal penetration of the epidermal and dermal layers is incomplete.

### 3.2 Future perspectives – a call to action

The time has come to move beyond the traditional approaches that have been used thus far. Our assessment is that the current limitations are merely technical hurdles, and not fundamental flaws. The ongoing developments are rapidly addressing these issues. In order to facilitate advancement within this field, it is crucial to acknowledge the importance of allocating resources towards resolving these challenges rather than attempting to circumvent them. To facilitate the next stages of development, it is essential that the required financial support, in form of specific investments, be provided by third-party funding entities.

We firmly believe that 3D epithelial models represent not merely an enhancement, but a vital progression in the field of *Candida* infection and treatment research. The potential benefits of this approach outweigh the existing obstacles and challenges. The limitations discussed in the preceding chapter indicate the necessary steps for the development of enhanced 3D models that more closely resemble the human epithelia *in vitro*. Standardized protocols for the construction and cultivation of 3D models, as well as for medium composition, need to be implemented across laboratories for the study of fungal infection and the performance of antifungal drug testing. The development of immune-competent 3D models has to be forced. The relatively high production costs can be mitigated through the implementation of standardization and automation. It is imperative to establish high-throughput screening protocols. The development of 3D bioprinting techniques offers the potential for a wider range of applications, including “skin-on-chip” technology, which enables high-throughput screening, real-time process monitoring, and the use of AI-integrated and automated analysis platforms (Jia and Atwood, 2024; Rhee et al., 2024). Additionally, 3D bioprinting technology provides unparalleled potential for the creation of intricate, bespoke skin tissue architectures, allowing the reconstruction of more precise and physiologically representative 3D models that can replicate a spectrum of pathological conditions and facilitate advanced research in *Candida* infection and antifungal drug development. Importantly, we highly advocate for a mandatory *in vitro* validation of new antifungal compounds using 3D skin models before animal testing.

It is also important to consider the potential of 3D epithelial models in investigating diseased skin conditions and their high predisposition to microbial superinfections, including *C. albicans*, due to specific skin inflammation or immune dysregulation conditions, such as those observed in atopic dermatitis, ichtyosis vulgaris, or psoriasis (Leman et al., 2019; Moosbrugger-Martinz et al., 2021). 3D models of diseased skin have significant potential in personalized medicine. When constructed with patient-specific cells, these models can be used to study the response to *C. albicans* infection under the respective genetic predisposition and pathophysiology, as well as to predict treatment response. Furthermore, drug-resistant *C. albicans* laboratory-generated mutant strains can be employed for the investigation of infection processes (Zakikhany et al., 2007), and clinical isolates for the identification of promising novel antifungal treatment options aimed at overcoming fungal resistance.

Finally, we want to attract the attention to another significant research area that has emerged in recent years: the study of the complex ecosystem of microorganisms that colonize human epithelia. The microflora of the human body comprises harmless commensals, including bacteria and fungi, which may potentially convert into pathogens under certain circumstances. It is imperative that the study of the human epithelial microbiome, the triggers that induce the switch from a commensal to a pathogenic life-style, the investigation of multi-species infection, and the identification of novel treatment options be implemented. This represents a relatively new but rapidly evolving field of research.
